# Clinical utility of circulating miR-371a-3p for the management of patients with intracranial malignant germ cell tumors

**DOI:** 10.1093/noajnl/vdaa048

**Published:** 2020-04-13

**Authors:** Matthew J Murray, Thankamma Ajithkumar, Fiona Harris, Rachel M Williams, Ibrahim Jalloh, Justin Cross, Milind Ronghe, Dawn Ward, Cinzia G Scarpini, James C Nicholson, Nicholas Coleman

**Affiliations:** 1 Department of Pathology, University of Cambridge, Cambridge, UK; 2 Department of Paediatric Haematology and Oncology, Cambridge University Hospitals NHS Foundation Trust, Cambridge, UK; 3 Department of Medical Oncology, Cambridge University Hospitals NHS Foundation Trust, Cambridge, UK; 4 Department of Paediatric Endocrinology, Cambridge University Hospitals NHS Foundation Trust, Cambridge, UK; 5 Department of Neurosurgery, Cambridge University Hospitals NHS Foundation Trust, Cambridge, UK; 6 Department of Neuroradiology, Cambridge University Hospitals NHS Foundation Trust, Cambridge, UK; 7 Department of Paediatric Haematology and Oncology, Royal Hospital for Children, Glasgow, UK; 8 Department of Histopathology, Cambridge University Hospitals NHS Foundation Trust, Cambridge, UK

**Keywords:** biomarker, germ cell tumor, microRNA, miR-371a-3p, vinblastine

## Abstract

**Background:**

The current biomarkers alpha-fetoprotein (AFP) and human chorionic gonadotropin (HCG) have limited sensitivity/specificity for diagnosing malignant germ cell tumors (GCTs) and “marker-negative” patients require histological confirmation for diagnosis. However, GCTs at intracranial sites are surgically relatively inaccessible and biopsy carries risks. MicroRNAs from the miR-371~373 and miR-302/367 clusters are over-expressed in all malignant GCTs and, in particular, miR-371a-3p shows elevated serum levels at diagnosis for testicular disease.

**Methods:**

Using our robust preamplified qRT-PCR methodology, we quantified miR-371a-3p levels in serum and cerebrospinal fluid (CSF) in a series of 4 representative clinical cases, 3 with intracranial malignant GCT and 1 with Langerhans cell histiocytosis (LCH), compared with appropriate control cases.

**Results:**

Serum and/or CSF miR-371a-3p levels distinguished those with intracranial malignant GCTs from LCH and, if known in real time, could have helped clinical management. The benefits would have included (1) the only confirmatory evidence of an intracranial malignant GCT in 1 case, supporting clinical decision making; (2) early detection of intracranial malignant GCT in another, where an elevated CSF miR-371a-3p level preceded the histologically confirmed diagnosis by 2 years; and (3) confirmation of an intracranial malignant GCT relapse with an elevated serum miR-371a-3p level, where serum and CSF AFP and HCG levels were below thresholds for such a diagnosis.

**Conclusions:**

This series highlights the potential for microRNA quantification to assist the noninvasive diagnosis, prognostication, and management for patients with intracranial malignant GCTs. Serum and CSF should be collected routinely as part of future studies to facilitate the extension of these findings to larger patient cohorts.

**Key Points**

- The majority of intracranial GCTs require biopsy as most are AFP/HCG marker-negative.
- Knowledge of serum/CSF miR-371a-3p microRNA levels could help clinical management.
- Serum and CSF should be collected routinely as part of future studies.

**Importance of the Study:**

The current biomarkers alpha-fetoprotein (AFP) and human chorionic gonadotropin (HCG) have limited sensitivity/specificity for diagnosing malignant germ cell tumors (GCTs). As most intracranial malignant GCTs are AFP/HCG marker-negative, the majority of patients undergo biopsy to establish the diagnosis. However, GCTs at intracranial sites are relatively surgically inaccessible and biopsy carries risks. MicroRNAs from the miR-371~373 and miR-302/367 clusters are over-expressed in all malignant GCTs and, in particular, miR-371a-3p shows elevated serum levels at diagnosis for testicular disease. Here, we showed that serum and/or cerebrospinal fluid (CSF) miR-371a-3p levels distinguished those with intracranial malignant GCTs from other tumors, and if known in real time, could have helped clinical management. This series highlights the potential for microRNA quantification to assist the noninvasive diagnosis, prognostication, and management for patients with intracranial malignant GCTs. Serum and CSF should be collected routinely as part of future studies to facilitate the extension of these findings to larger patient cohorts.

Germ cell tumors (GCTs) are characterized by clinical and pathological heterogeneity. Malignant GCTs are classified into germinoma and non-germinomatous tumors (yolk sac tumor [YST], embryonal carcinoma [EC], and choriocarcinoma [CHC]), while teratomas (which show extensive somatic differentiation) are generally considered benign.^1^ Their anatomical occurrence at both gonadal and extragonadal (including intracranial) sites adds to their complexity and diagnosis may be challenging, particularly for extragonadal/intracranial cases, which are surgically relatively inaccessible. The utility of the conventional body fluid protein biomarkers alpha-fetoprotein (AFP) and human chorionic gonadotropin (HCG) for diagnosis and follow-up is restricted to specific malignant GCT subtypes, as levels are raised predominantly in tumors containing YST and CHC, respectively.^2^ For extragonadal primary sites of disease (eg, retroperitoneum, mediastinum, and the central nervous system [CNS]), typical radiological findings and raised AFP/HCG markers alone may be sufficient for diagnosis, and indeed for intracranial GCTs, this is the recommended practice in Europe and North America.^3^ For “marker-negative” extragonadal/intracranial cases, however, a biopsy is required to establish a formal histopathological diagnosis; this includes the majority of intracranial GCT cases, where germinomas (which are “nonsecreting”)^4^ occur approximately twice as frequently as non-germinomas.^5^ Such procedures carry risks of morbidity, due to the difficulties in surgical access to these anatomical sites. Consequently, body fluid biomarkers that offer greater sensitivity and specificity for diagnosing and/or monitoring malignant GCTs would be of major clinical benefit.^6^

Recent years have seen the emergence of circulating microRNAs as a new generation of biomarkers in malignant GCTs.^7^ MicroRNAs are short, non-protein-coding RNAs that are highly stable and well-suited for disease diagnosis and monitoring.^8^ We previously demonstrated that miRNAs from the miR-371~373 and miR-302/367 clusters were over-expressed in all malignant GCT tissues^1^ and developed a highly sensitive preamplified qRT-PCR protocol to identify that levels of these microRNAs were elevated in patient serum at the time of malignant GCT diagnosis,^9^ with a panel of 4 serum microRNAs (miR-371a-3p, miR-372-3p, miR-373-3p, and miR-367-3p) highly sensitive and specific for this purpose.^10–12^ Of note, miR-371a-3p appears to show the greatest predictive value from the panel.^13–18^ We have shown that serum microRNA levels fall with successful treatment and increase rapidly at the time of relapse.^9,11^ We have also demonstrated that levels of these microRNAs were elevated in the serum and/or cerebrospinal fluid (CSF) at the time of diagnosis of intracranial malignant GCTs.^11^ Multiple international research teams have adopted our preamplified methodology and demonstrated the utility of circulating microRNA testing for patients with malignant GCTs, but almost exclusively in adult male patients with testicular disease.^12–14,18,19^ Two clinical case series have highlighted how testicular GCT management could have been altered if knowledge of the circulating microRNA results were available in real time.^20,21^ The present case series, with associated long-term follow-up and outcomes, demonstrates how knowledge of circulating (serum and/or CSF) microRNA results for patients with a differential diagnosis that includes intracranial GCT could facilitate clinical management. Further work is now justified to establish these microRNAs in clinical practice, not just for patients with testicular disease^16^ but also for those with extragonadal disease, including intracranial cases.^22^

## Materials and Methods

The study was performed under Cambridge Local Research Ethics Committee (reference 01/128) and the generic Children’s Cancer and Leukaemia Group (CCLG) Tissue Bank (reference 08/h0405/22 + 5, covering CCLG Biological Study 2002 BS 03) approvals and was performed with full informed parental consent. All experimental steps were compliant with the Minimum Information for Publication of Quantitative Real-time PCR Experiments.^23^ Sample processing, RNA isolation, and microRNA quantification were performed as described.^11,24^ In short, as part of each patient’s standard clinical care, blood was sampled in serum separator tubes and/or CSF collected in plain tubes, before processing and centrifugation within 4 h of receipt.^11^ After routine clinical measurements, samples were kept at 4^o^C before residual serum/CSF was frozen and stored at −80^o^C,^11^ prior to processing. A set quantity of the exogenous non-human spike-in cel-miR-39-3p was added to serum/CSF prior to RNA extraction and used in an initial quality control check to measure RNA extraction efficiency, as described.^11,24^ A multiplexed reverse transcription step was then performed followed by a multiplexed preamplification step, maximizing assay sensitivity,^9,11,24^ prior to a final singleplexed PCR of the diluted preamplification product and then normalization to the endogenous stable housekeeping microRNA miR-30b-5p,^11^ in order to obtain relative miR-371a-3p levels.

## Case Presentations and Results

### Case 1

**Figure 1.**
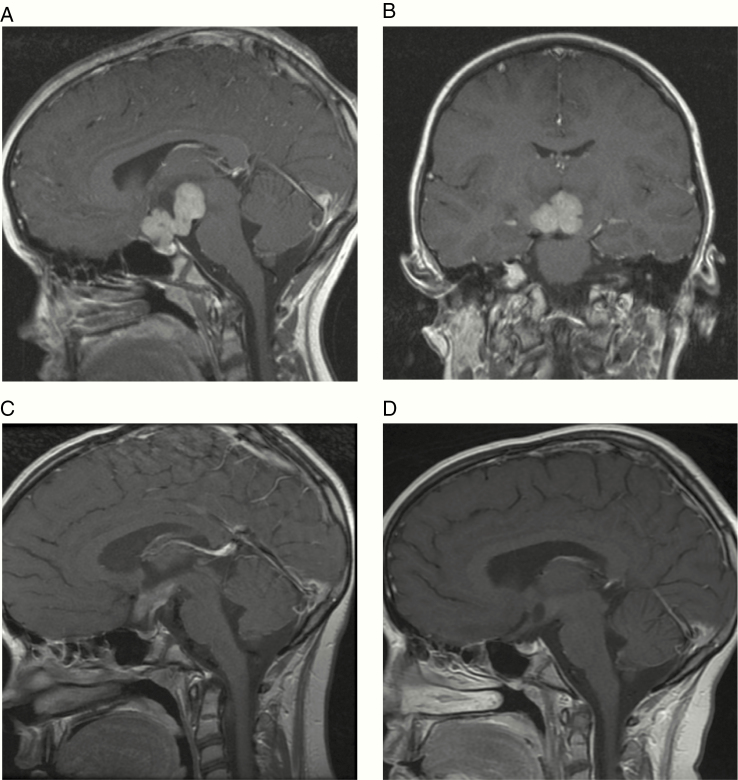
MRI of Case 1 (suprasellar germinoma). (A and B) representative sagittal and coronal views, respectively, at the time of presentation. (C) sagittal view after 2 doses of weekly vinblastine showing dramatic tumor response and (D) sagittal view after completion of vinblastine monotherapy and prior to definitive radiotherapy showing further tumor response.

**Figure 2.**
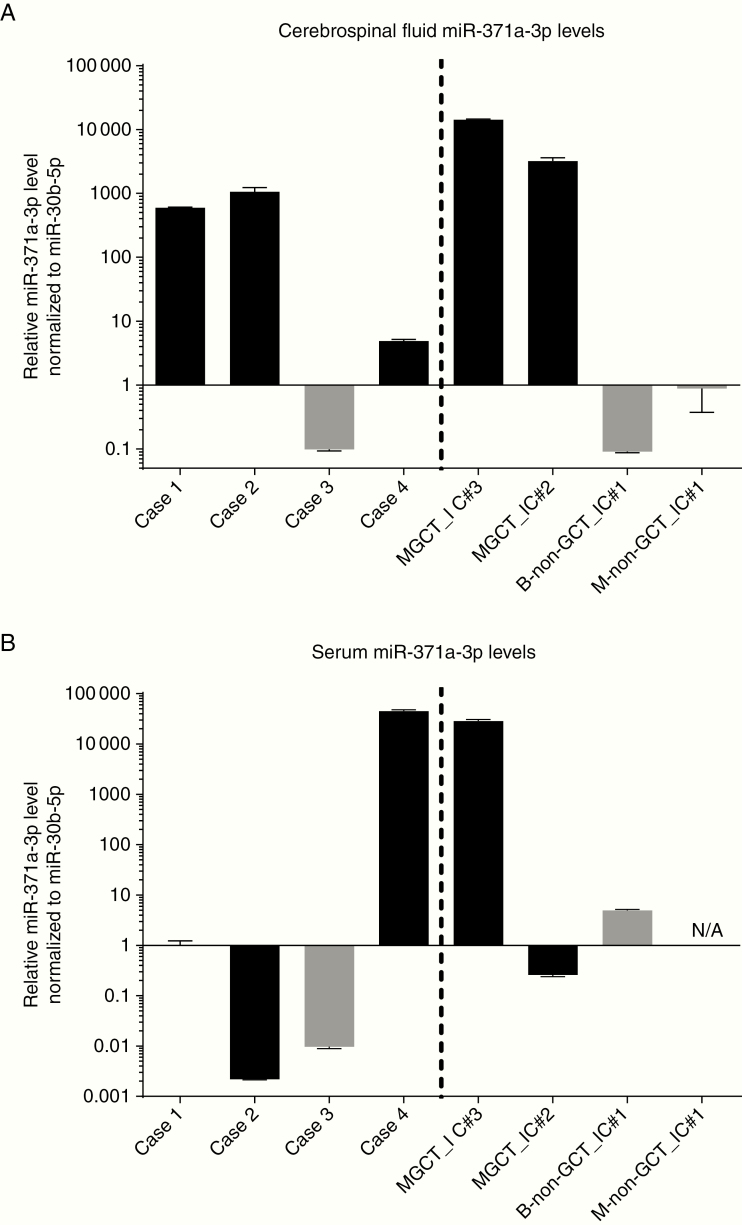
Relative miR-371a-3p levels in cerebrospinal fluid (CSF) and serum from the cases. (A) CSF and (B) serum miR-371a-3p levels in Cases 1–4, compared with 4 previously published cases.^11^ The published cases comprised 2 positive controls (intracranial suprasellar malignant GCT cases, MGCT_IC#3 and MGCT_IC#2) and 2 negative controls (a suprasellar low-grade glioma case, B-non-GCT_IC#1, and a patient with intracranially disseminated high-risk neuroblastoma).^11^ N/A, not available. Error bars represent standard error of the mean.

A 9-year-old girl presented to the ophthalmologist with a 2-year history of worsening vision. She was also noted to have longstanding polyuria and polydipsia consistent with diabetes insipidus (DI). On examination, uncorrected near and distance visual acuity revealed minimal/no vision in the right eye and severely affected vision in the left eye. Severe bilateral optic atrophy was noted, worse in the right eye. MRI head scan revealed a 36 mm diameter lobulated enhancing suprasellar solid mass involving the optic chiasm (Figure 1A and B). Initially, the lesion was thought to be most consistent with low-grade glioma (LGG) and single-agent vinblastine monotherapy was initiated without biopsy. The patient’s vision returned within 4 days. Neuro-oncology multidisciplinary team (MDT) discussion raised the possibility of a malignant GCT (germinoma) or LGG and complete work-up and staging were recommended. Tumor markers (serum and CSF AFP and HCG) and CSF cytology, performed after 2 doses of weekly vinblastine had been delivered, were negative. Remaining CSF from the procedure and leftover serum were also stored for microRNA quantification. Repeat MRI at this time, with spinal imaging, performed 22 days after the first scan, revealed a substantial decrease in size and enhancement of the suprasellar mass, which was now 21 x 19 mm in axial diameter (Figure 1C) compared with 36 x 28 mm originally. No new lesions were demonstrated, and spinal MRI was normal. A neurosurgical biopsy was discussed but given the dramatic clinical and radiological response after just 2 doses of vinblastine, it was determined that biopsy would be challenging and moreover unlikely to be diagnostic. Quantification of miR-371a-3p levels at this time showed a value of 1.00 in the serum but an elevated level of 596 in the CSF (Figure 2), consistent with CSF levels observed in proven intracranial malignant GCTs.^11^ Initiation of carboPEI chemotherapy (carboplatin/etoposide alternating with etoposide/ifosfamide), standard pre-radiotherapy chemotherapy treatment for germinoma,^4^ was discussed with the patient’s family. However, due to the remarkable early response obtained, and for religious reasons, they wished to avoid the additional side effects, particularly relating to bone marrow suppression, expected with standard carboplatin-based therapy, but rather to continue vinblastine, which has good CNS penetration and is well tolerated.^25–27^ Weekly vinblastine monotherapy was therefore continued and further re-assessment scan showed continued response with a further decrease in size and enhancement of the suprasellar lesion (Figure 1D), prior to definitive radiotherapy. In total, 12 doses of vinblastine were delivered across 3 months. Given full staging was not performed prior to the commencement of chemotherapy, it was determined that craniospinal irradiation (CSI) would be the most appropriate treatment. The initial plan was to deliver 24 Gy CSI followed by a 16 Gy boost, in keeping with current European practice.^4^ However, the patient’s family was concerned about long-term sequelae from this dose. Since the MRI scan after single-agent vinblastine showed excellent response, it was determined that the North American practice of 21 Gy CSI followed by 9 Gy boost would likely be sufficient to ensure a high cure rate.^28–30^ The patient proceeded to radiotherapy and remains well in follow-up, with panhypopituitarism, as expected, 48 months from initial presentation and diagnosis. The patient remains on regular MRI surveillance, with scans since the end of treatment showing a stable, non-enhancing residual suprasellar mass measuring 14 mm × 12 mm diameter on axial imaging, of no prognostic significance.^4^

### Case 2

**Figure 3.**
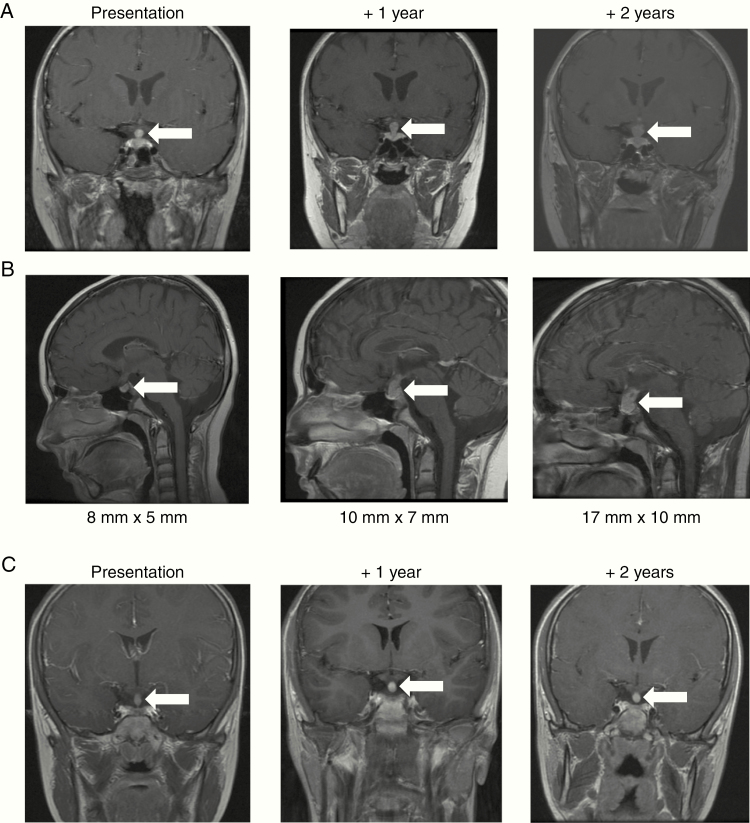
MRI of Cases 2 and 3 presenting with pituitary thickening and diabetes insipidus. Representative (A) coronal views of Case 2, (B) sagittal views of Case 2, and (C) coronal views of Case 3—at first presentation (left panels), 1 year later (central panels), and 2 years later (right panels). White arrows indicate the radiological abnormality.

A 15-year-old boy presented to the pediatric endocrinology team with a 4-year history of polydipsia and polyuria characteristic of DI. In addition, over the previous year he had developed short stature which on investigation revealed low growth hormone levels. He also had evidence of delayed puberty; bone age on wrist X-ray was delayed by 4–5 years. MRI of the pituitary gland demonstrated a thickened (5 mm maximum transverse diameter), enhancing pituitary region/infundibulum (Figure 3A). The high signal (“bright spot”) from the posterior pituitary lobe was noted to be absent. The report stated that Langerhans cell histiocytosis (LCH) or a malignant GCT (germinoma) was the most likely diagnosis, a view confirmed at the subsequent neuro-oncology MDT meeting. Full staging work-up was performed for both these conditions, which was negative. In particular, MRI head and spine showed no other lesions, and serum and CSF AFP and HCG and CSF cytology were negative. Remaining CSF from the lumbar puncture and leftover serum was stored for microRNA quantification. In summary, following comprehensive work-up, there was no diagnostic evidence of either active LCH or germinoma. Neurosurgical biopsy of the pituitary thickening was discussed at the MDT, but at 5 mm diameter, it was determined that biopsy would likely have low diagnostic yield and further pituitary damage was likely. Consequently, the patient entered an MRI surveillance schedule, initial 6-monthly, with the patient and his family aware that if there was a persistent increase in the size of the lesion, a further work-up would be required. Surveillance MRIs 1 and 2 years later revealed that the pituitary stalk was 7 mm and 10 mm in diameter, respectively (Figure 3A). On sagittal views, the lesion had increased from 8 mm (craniocaudally) × 5 mm (anterior–posterior) diameter initially through 10 mm × 7 mm at 1 year to 17 mm × 10 mm at 2 years (Figure 3B). In view of this, further marker (AFP/HCG) and CSF cytology examinations were performed, which were negative. A biopsy was therefore undertaken which revealed a prominent lymphocytic infiltrate, within which was a clear population of large cells with large nuclei and prominent nucleoli, with positive co-staining for membranous PLAP and CD117 (KIT) and nuclear POU5F1 (OCT3/4), consistent with germinoma. The patient was consented onto the European SIOP-CNS-GCT-II trial and a further MRI was performed prior to initiation of carboPEI chemotherapy. This showed a 20 mm × 14 mm heterogeneously enhancing solid and cystic suprasellar tumor but no other changes. The patient started treatment 28 months following the initial hospital presentation. Following chemotherapy, the pituitary lesion had reduced substantially in size to 13 mm × 6 mm diameter. After radiotherapy (24 Gy focal and whole ventricular irradiation, plus 16 Gy tumor boost), the pituitary lesion could no longer be visualized on MRI. The patient remains well 54 months from the initial presentation and 20 months following the completion of treatment. MRI scans remain clear and the patient, now 20 years old, continues with oncological and endocrine follow-up. Of particular interest, while the serum miR-371a-3p level was uninformative, the miR-371a-3p level performed on the available CSF sample when the patient first presented at 15 years of age was elevated, at a relative level of 1062 (Figure 2), consistent with CSF levels observed in proven intracranial malignant GCTs.^11^

### Case 3

A 12-year-old girl was being followed for a 6-year history of DI and growth hormone deficiency. She had undergone serial MRI scans over a number of years which had shown stable mild thickening (3 mm AP diameter) of the pituitary stalk, and routine MRIs had therefore been discontinued, and a presumptive diagnosis of LCH made. New symptoms (hearing loss and ear discharge) resulted in a further MRI being performed which showed that the pituitary stalk had increased to 5 mm diameter over the 3-year period since the previous scan (Figure 3C) but nil else of note. Full work-up for active LCH and germinoma, as previously described, was negative and remaining CSF and leftover serum were stored for microRNA quantification. Serum and CSF miR-371a-3p levels were normal at 0.01 and 0.099, respectively (Figure 2), consistent with levels observed in control patients.^11^ Serial MRI over the following years revealed no change in the enhancing pituitary stalk thickening, which remained stable at 5 mm diameter (Figure 3C). Consistent with the presumptive diagnosis of LCH, the patient subsequently developed other LCH-associated changes on MRI, with abnormal intensity in the dentate nuclei bilaterally and adjacent cerebellar white matter, but remained otherwise clinically well and therefore did not require treatment. The patient is now 53 months following re-presentation, remains well, and continues with endocrinology follow-up and annual MRI scans.

### Case 4

**Figure 4.**
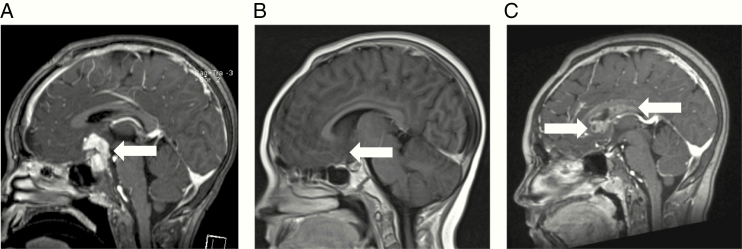
MRI of Case 4 with germinoma relapse of an intracranial malignant non-germinomatous germ cell tumor (NGGCT). Representative sagittal views at (A) original diagnosis of the NGGCT, (B) following completion of treatment with chemotherapy and radiotherapy, and (C) at the time of relapse with germinoma, with nodular enhancing cyst-like lesions posterior and inferior to the corpus callosum and involving the septum pellucidum. White arrows indicate the radiological abnormality.

An 8-year-old girl presented with symptoms of raised intracranial pressure and DI. MRI scan showed an enhancing suprasellar mass measuring 33 mm × 29 mm (Figure 4A). She underwent a neurosurgical procedure which histologically confirmed a mixed malignant non-germinomatous GCT (NGGCT), containing both YST (consistent with her serum and CSF AFP levels of 198 and 2695 kU/L, respectively) and germinoma components. Serum and CSF HCG were both normal (<2 IU/L). Staging with MRI spine and CSF cytology showed localized disease only. She underwent treatment according to the guidelines following the closure of the European SIOP-CNS-GCT-96 trial, with 4 courses of intensive PEI chemotherapy (cisplatin, etoposide, and ifosfamide) followed by focal 54 Gy radiotherapy to the pituitary area only.^5^ MRI imaging at that stage showed a good response with only a small non-enhancing 9 mm residual that was not felt to be resectable (Figure 4B). At 11 years of age, and 3 years following the end of treatment, routine surveillance MRI demonstrated an asymptomatic relapse outside the radiotherapy field, with nodular enhancing cyst-like lesions posterior and inferior to the corpus callosum and involving the septum pellucidum (Figure 4C), measuring 43 mm × 19 mm × 11 mm. Spinal imaging and CSF cytology were clear. Repeat AFP tumor markers were negative (serum 8 kU/L and CSF 1 kU/L). Serum and CSF HCG demonstrated very slightly raised levels of 4 IU/L and 16 IU/L (reference range <2 IU/L), respectively, but below the threshold of 50 IU/L to be considered “secreting” on the European trial protocols (SIOP-CNS-GCT-96 and SIOP-CNS-GCT-II) and guidelines.^5^ Remaining CSF and leftover serum were stored for microRNA quantification. A biopsy was performed, which confirmed germinoma only, consistent with the very mildly elevated HCG levels (due to HCG production from syncytiotrophoblast cells within the germinoma^2^). Serum and CSF miR-371a-3p quantification at the time of relapse revealed levels of 44 870 and 4.88, respectively (Figure 2), consistent with levels observed in patients with proven intracranial malignant GCTs.^11^ The patient underwent re-induction chemotherapy with carboplatin/etoposide as per the German HIT-REZ protocol followed by consolidation with high-dose chemotherapy and autologous stem cell rescue, as recommended for NGGCT relapses.^31^ Having only had focal radiotherapy for primary treatment, CSI was then delivered, adding onto the original focal field. The patient remains well, with MRI scans showing no evidence of disease, 51 months from the time of relapse and 42 months from the end of treatment.

## Discussion

The clinical management of GCTs is complex as they are heterogeneous with regard to disease site and histological subtype(s).^1^ Furthermore, the limited sensitivity of the current serum and CSF markers AFP and HCG restricts their clinical utility for the management of malignant GCTs.^2^ AFP is typically raised in the YST subtype and HCG in CHC, but these markers are usually negative in germinoma/seminoma and EC subtypes. A universal biomarker of all malignant GCTs would offer many benefits^2,15–17^ and circulating microRNAs are likely to answer this unmet clinical need.^16^

MicroRNAs are short, non-protein-coding RNAs that regulate the expression of protein-coding genes and thereby critical cellular processes.^1^ Importantly, dysregulated microRNA expression profiles classify human cancers.^32^ Malignant GCTs are universally characterized by high expression levels of all 8 microRNA members of the miR-371~373 and miR-302/367 clusters, regardless of patient age, tumor site, or histological subtype.^1^ Importantly, this co-ordinate over-expression does not occur in any other tumor type or disease state,^1^ suggesting these microRNAs could offer high specificity for malignant GCTs. Here, we detail 4 intracranial patient cases with long-term outcome data, demonstrating how knowledge of circulating microRNA results for those with a differential diagnosis including intracranial GCT could in future facilitate real-time clinical management and highlighting important clinical messages.

For Case 1, with the suprasellar mass, DI and visual loss, the clinical history and radiological findings were initially interpreted as representing an LGG and the patient commenced on vinblastine monotherapy.^25,26^ The dramatic and rapid reversal of visual loss within days meant that the provisional diagnosis was widened to include a malignant GCT (germinoma).^33^ Re-assessment MRI after 2 vinblastine doses showed such a dramatic response (Figure 1C) that the risks of neurosurgical biopsy were felt to outweigh any potential benefits, particularly given the expected nondiagnostic outcome. Therefore, with normal serum and CSF AFP and HCG and negative CSF cytology at that stage, the only evidence for an intracranial germinoma was the highly elevated CSF miR-371a-3p level at the time of this lumbar puncture (Figure 2), which supported the clinical decision to give CSI following vinblastine monotherapy. Interestingly, although carboPEI, consisting of combinations of etoposide, with alternating carboplatin and ifosfamide, is the standard European pre-irradiation chemotherapy schedule for patients with a localized intracranial germinoma in order to reduce radiotherapy fields,^4^ the response to vinblastine monotherapy is particularly noteworthy and consistent with early use of carboplatin monotherapy to reduce radiotherapy doses.^34^ Vinblastine monotherapy is well tolerated with minimal side effects for the treatment of other CNS conditions such as LGG and LCH.^25–27^ Potential use of vinblastine for intracranial germinoma, prior to definitive radiotherapy, could be explored in future studies, particularly as an international consensus process confirmed that the current overall aim of management for this malignant GCT subtype is to maintain excellent overall survival rates while attempting to minimize late effects of treatment.^33^ Avoiding the concomitant intravenous hydration and prolonged inpatient admissions associated with current chemotherapy schedules, which can exacerbate preexisting DI, would be another particularly attractive characteristic of weekly vinblastine, typically delivered as an intravenous bolus in an outpatient setting.

The clinical conundrum of managing patients with pituitary thickening and DI was exemplified by Cases 2 and 3, where the 2 patients had almost identical clinical and radiological presentations (Figure 3). Diagnostic work-up for active LCH and germinoma in such cases is challenging and suboptimal. The low sensitivity of current diagnostic work-up means that negative results do not necessarily exclude such diagnoses. For example, the majority of germinomas are AFP/HCG “marker-negative” and as they are only metastatic in less than 20% of cases (by imaging and/or cytology),^4^ CSF cytology alone has a very low diagnostic yield. Furthermore, the potential risks of neurosurgical biopsy for 5 mm pituitary thickening were considered excessive in both cases, due to the risks of causing further hormonal disturbance and the perceived low diagnostic yield. Consequently, a surveillance imaging program was instituted for both patients. The CSF miR-371a-3p results obtained in these patients were completely different (Figure 2). Case 2 had a highly elevated level and the pituitary thickening progressed for 2 years before germinoma was confirmed. In contrast, Case 3 had a normal level and the pituitary thickening remained stable, as expected, on radiological imaging. These data suggest that quantifying microRNA levels could be used to dichotomize such patients and facilitate early detection of intracranial germinoma. An elevated CSF level, consistent with those observed in proven intracranial malignant GCTs,^11^ could be used to re-consider early neurosurgical biopsy, whereas a normal level would provide reassurance to continue a surveillance program and observe for clinical features of active LCH. This is of particular clinical relevance, as a prolonged symptom interval (SI) occurs in one-third of intracranial GCT patients and is associated with an increased risk of metastatic disease.^35^ As metastatic disease requires treatment with CSI and is associated with increased treatment burden and late effects,^35^ interventions to reduce SI, such as early microRNA quantification in cases of pituitary thickening and DI, should be explored further.

Case 4, a patient with intracranial NGGCT relapsing with germinoma component (Figure 4), serves to highlight the importance, just as with AFP/HCG,^33^ of measuring microRNA levels in both the serum and CSF compartments. While the CSF miR-371a-3p was only 4.88, the serum level was substantially elevated at 44 870, consistent with serum levels observed in another patient with an intracranial malignant GCT (Figure 2). Such observations are likely to relate to intracranial site, tumor proximity to blood vessels, and local blood flow and warrant further study. Future biospecimen collection therefore needs to include a collection of serum and CSF to facilitate such studies.^22^

Ultimately, assisted by such biospecimen collections, a future aim could be for completely noninvasive diagnosis of, and prognostication for, intracranial GCTs. Biopsy for intracranial GCTs is challenging due to the central, midline nature of the majority of the lesions, which predominantly occur at suprasellar and/or pineal sites, and risk of complications include hemorrhage, infection, stroke, and death.^3^ Indeed, a previous case of histologically confirmed intracranial suprasellar germinoma we reported with elevated CSF miR-371a-3p levels, developed DI postoperatively, causing disturbed sodium homeostasis and seizures requiring intensive care admission.^11^ In the future, quantification of miR-371a-3p in the serum and CSF will facilitate the diagnosis of a malignant GCT and exclude other CNS tumors. However, that alone will be insufficient for prognostication and treatment risk stratification, as patients with intracranial GCTs need to be segregated into those with germinoma and those with NGGCT, as the latter needs a more intensive chemotherapy and radiation strategy due to their relatively inferior outcomes.^5^ A specific challenge is that intracranial GCTs are often mixed tumors, containing more than one histological component. Any strategy for the noninvasive diagnosis and prognostication for intracranial malignant GCTs therefore needs to be able to accurately identify the presence of the most aggressive histological subtype. In practice, as thresholds for AFP and HCG levels can be employed pragmatically for detecting the malignant NGGCT components YST and CHC, respectively,^5^ the main clinical priority is for a second noninvasive microRNA panel that could distinguish the typically AFP/HCG “marker-negative” malignant GCT subtypes germinoma/seminoma versus EC. At present, although the majority of such biopsied cases show germinoma alone, it is important to identify the EC subtype as its presence requires more intensified NGGCT treatment.^5^

In summary, we have utilized this case series to demonstrate the clinical value of circulating (CSF and serum) microRNA quantification for the management of patients with intracranial malignant GCTs. Appropriate biospecimen collection, including serum and CSF, needs to be embedded in future clinical trials,^22^ in order to facilitate progress toward noninvasive diagnosis, prognostication, and management of these tumors.
